# Distinct effects of rs895819 on risk of different cancers: an update meta-analysis

**DOI:** 10.18632/oncotarget.17454

**Published:** 2017-04-27

**Authors:** Muxiong Chen, Wenpan Fang, Xinkai Wu, Suchen Bian, Guangdi Chen, Liqin Lu, Yu Weng

**Affiliations:** ^1^ Institute of Environmental Health, Zhejiang University School of Medicine, Hangzhou, China; ^2^ Research Center of Molecular Medicine, Zhejiang University School of Medicine, Hangzhou, China; ^3^ Department of Public Health, Zhejiang University School of Medicine, Hangzhou, China; ^4^ Department of Oncology, Zhejiang Provincial People’s Hospital, Hangzhou, China; ^5^ Department of Clinical Laboratory, Sir Run Run Shaw Hospital, Zhejiang University School of Medicine, Hangzhou, China

**Keywords:** miR-27a, genetic variant, cancer risk, meta-analysis

## Abstract

Previous studies have indicated an association between the genetic variant in pre-miR-27a rs895819 with A->G transition and cancer risk; however, the results remain inconsistent and somehow conflicting in different cancers. Therefore, to obtain a more reliable conclusion, we performed an update meta-analysis by searching PubMed database or other databases. Odds ratio (ORs) and 95% confidence interval (CIs) were calculated to evaluate cancer risk. A total of 34 case-control studies involving 15,388 cases and 18,704 controls were included. The results showed that rs895819 was associated with an increased cancer risk (GG *vs.* AA/AG: OR = 1.15, 95% CI = 1.02–1.29). Furthermore, stratification analyses revealed an association of rs895819 with increased cancer risk among Asians (GG *vs.* AA: OR = 1.17, 95% CI = 1.01–1.36; GG *vs.* AA/AG: OR = 1.18, 95% CI = 1.03–1.35), but not Caucasians. Interestingly, the [G] allele of rs895819 was significantly associated with decreased risk of breast cancer (G *vs.* A: OR = 0.91, 95% CI = 0.86–0.97). However, rs895819 was associated with increased risk of colorectal cancer (GG *vs.* AA: OR = 1.56, 95% CI = 1.31–1.85; GG *vs.* AA/AG: OR = 1.53, 95% CI = 1.30–1.79; G *vs.* A: OR = 1.19, 95% CI = 1.09–1.30) and lung cancer (GG *vs.* AA/AG: OR = 1.43, 95% CI = 1.00–2.04). In addition, no association was found between rs895819 and risk of gastric cancer or esophageal cancer. In conclusion, our findings suggest distinct effects of rs895819 on risk of different cancers, and future well-designed studies with large samples are required to further validate our results.

## INTRODUCTION

MicroRNAs (miRNAs) are 18–25 nucleotides length of noncoding single-stranded RNAs that post-transcriptionally silence gene expression through degradation of messenger RNA (mRNA) targets and (or) block protein translations of these targets [1]. MiRNA are widely expressed in the yeast, animal and plant genomes and have been implicated in many important physiologic and pathologic processes such as cell proliferation, differentiation, migration, autophagy and apoptosis, etc [2]. Dysregulation of miRNA expression has been found to have relevance not only to tumorigenesis, but also to neurological, cardiovascular, developmental and other diseases [3].

Single nucleotide polymorphisms (SNPs) are a type of polymorphism involving variation of a single base pair. Recent DNA sequencing has revealed SNPs in miRNA coding genes, both in miRNA seeding and loop regions [4, 5]. SNPs present in the miRNA gene regions can affect their transcription and maturation processing through their transcripts (pri-miRNA, pre-miRNA), which lead to aberrant mature miRNA expression levels [6]. In addition, SNPs in seeding regions of miRNA genes may influence miRNA-mRNA interactions and eventually alter functions of miRNAs on targets [6]. Accumulating studies showed that SNPs in miRNAs or their precursors are marked as novel genetic variations which may modify the cancer susceptibilities [7].

The oncogenic miR-27 is known to regulate pathogenesis in numerous types of cancer, including breast cancer, esophageal cancer, gastric cancer and lung cancer [3, 8–12]. Previously, a common single nucleotide polymorphism in pre-miR-27a, rs895819, has been demonstrated to be associated with decreased risk of breast cancer risk, but later studies showed conflicting associations [13–20]. Some other epidemiological studies indicated that rs895819 was associated with increased risk of gastric cancer, and the genotypes of rs895819 was correlated with miR-27a expression levels, however, other studies showed lack association of rs895819 with gastric cancer risk [21–27]. Meta-analysis studies revealed that rs895819 was a functional SNP and may have some relation to colorectal cancer susceptibility, especially in Asians [28]. Generally, the current available data were inconsistent about the effects of rs895819 on carcinogenesis in different cancers [28, 29], this discrepancy maybe partially attributed to the heterogeneity of the cancer subtype, small sample size, and ethnicity of the study population. Therefore, it is necessary to conduct a comprehensive review and meta-analysis of published data from all eligible studies on the association of rs895819 with cancer risk. In this study, we performed an update meta-analysis by including more recent publications to improve the efficiency and to drive a more precise estimation of the association between rs895819 SNP and cancer risks.

## RESULTS

### Characteristics of studies

**Figure 1:**
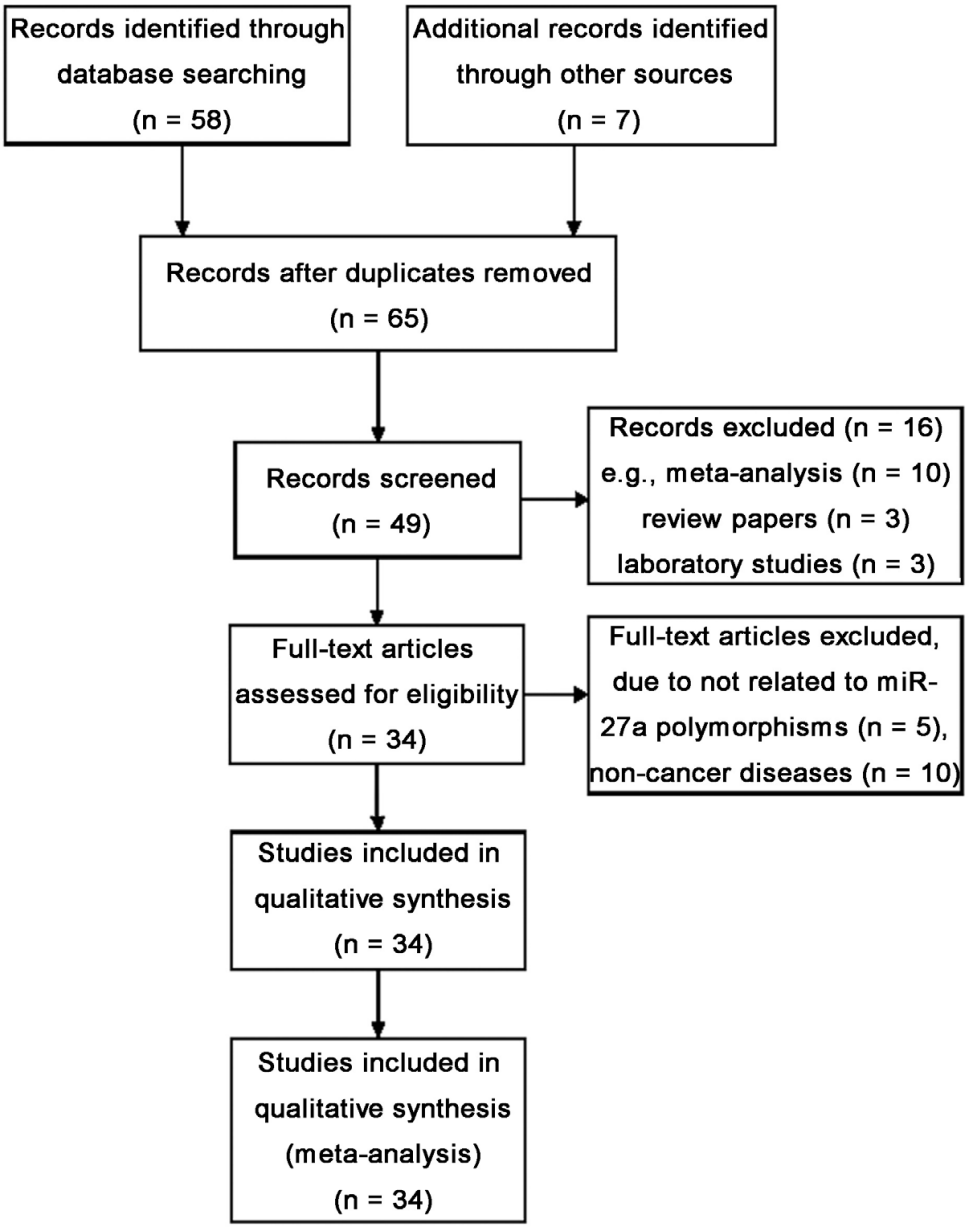
Flow diagram of studies identification

Fifty-eight abstracts were retrieved after the search “miR-27a”, “polymorphism” and “cancer”, and 27 articles were identified as eligible studies. Among the 58, 10 articles were pooled analysis, commentary [32–41] and 3 articles were review papers [28, 29, 42], and 3 reports were cancer biology experimental studies [43–45]. Ten studies were excluded because they reported non-cancer disease [46–55]. Five studies were excluded due to not related to miR-27a polymorphism, or no controls [56–60]. We also included 6 eligible articles by manual searching [16, 20, 25, 61–63], in which the study by Li *et al.* included two independent case-control studies [13] and the pooled analysis by Xu *et al.* presented efficient case-control study data. Totally, 34 eligible studies from 33 articles met the inclusion criteria were included in the meta-analysis (Figure 1).

Totally, 15,388 cases and 18,704 controls were included from 34 studies, including eight studies for breast cancer with 3,967 cases and 5,013 controls, eight for colorectal cancer with 2,381 cases and 3,058 controls, eight for gastric cancer with 4,016 cases and 4,782 controls, three for lung cancer with 1,284 cases and 1,393 controls, and two for esophageal cancer with 1,488 cases and 1,652 controls. For other cancers, there was only one study was included for each type of cancer, including live cancer [61], nasopharyngeal cancer [64], renal cancer [64], cervical cancer [65] and prostate cancer [66]. For ethnic distribution, there were twenty-seven studies of Asian origin, and seven studies on Caucasian descent. For the study design, the sources of controls from 8 studies were population-based, and the others were hospital-based. The genotype frequencies in the control group for each included study were consistent with HWE except three studies [14, 62, 67]. The selected study characteristics were listed in Table 1.

**Table 1:** Characteristics of studies included in the meta-analysis

Author	Year	Origin	Ethnicity	Cancer type	Sample size (case/control)	HWE	MAF	Design	Genotyping method
Yang R	2010	Germany	German	Breast cancer	1189/1416	0.142	0.340	PB	DNA Sequencingg
Zhang P	2011	China	Chinese	Breast cancer	376/190	0.605	0.258	PB	MassARRAY
Catucci I	2012	Italy	Italian	Breast cancer	1025/1593	0.051	0.297	PB	TaqMan
Zhang M	2012	China	Chinese	Breast cancer	245/243	0.122	0.467	PB	PCR-RFLP
Zhang N	2013	China	Chinese	Breast cancer	264/255	0.446	0.261	HB	TaqMan
Wang P	2014	China	Chinese	Breast cancer	107/219	0.537	0.237	HB	PCR-RFLP
Qi P	2015	China	Chinese	Breast cancer	321/290	0.686	0.433	PB	TaqMan
Morales S	2016	America	American	Breast cancer	440/807	0.017	0.280	HB	TaqMan
Sun Q	2010	China	Chinese	Gastric cancer	304/304	0.053	0.327	HB	PCR-RFLP
Zhou Y	2012	China	Chinese	Gastric cancer	295/413	0.941	0.280	HB	MALDI-TOF
Xu Q	2013	China	Chinese	Gastric cancer	222/305	0.437	0.252	HB	DNA Sequencingg
Yang Q	2014	China	Chinese	Gastric cancer	592/978	0.517	0.383	PB	TaqMan
Kupcinskas J	2014	Latvia	Lithuanian	Gastric cancer	363/350	0.151	0.320	HB	TaqMan
Song B	2014	China	Chinese	Gastric cancer	278/278	0.110	0.329	HB	TaqMan
Jiang J	2016	China	Chinese	Gastric cancer	895/988	0.447	0.260	HB	MassARRAY
Xu Q	2017	China	Chinese	Gastric cancer	1067/1166	0.161	0.247	HB	MALDI-TOF MS
Zhang M	2012	China	Chinese	Colorectal cancer	463/468	0.351	0.246	PB	PCR-RFLP
Hezova R	2012	Czech	Caucasian	Colorectal cancer	197/212	0.867	0.340	HB	TaqMan
Wang Z	2014	China	Chinese	Colorectal cancer	205/455	2.156	0.524	HB	TaqMan
Kupcinskas J	2014	Latvia	Lithuanian	Colorectal cancer	191/428	0.235	0.303	HB	TaqMan
Cao Y	2014	China	Chinese	Colorectal cancer	254/238	0.089	0.326	HB	PCR–RFLP
Wu R	2014	China	Chinese	Colorectal cancer	151/283	0.016	0.201	HB	DNA Sequencingg
Bian Q	2015	China	Chinese	Colorectal cancer	412/412	0.389	0.301	HB	TaqMan
Jiang Y	2016	China	Chinese	Colorectal cancer	508/562	0.053	0.313	HB	TaqMan
Wei J	2013	China	Chinese	Esophageal Cancer	379/377	0.322	0.264	HB	MALDI-TOF MSS
Zhang J	2014	China	Chinese	Esophageal Cancer	1109/1275	0.226	0.253	PB	PCR
Ma J Y	2015	China	Chinese	Lung cancer	542/557	0.015	0.308	HB	TaqMan
Yin Z	2015	China	Chinese	Lung cancer	167/228	0.282	0.228	HB	TaqMan
Yin Z	2016	China	Chinese	Lung cancer	575/608	0.199	0.270	HB	TaqMan
Li P	2011	China	Chinese	Nasopharyngeal Cancer	801/1022	0.658	0.295	HB	SNP Stream
Shi D	2011	China	Chinese	Renal cancer	594/600	0.373	0.302	HB	TaqMan
Li P	2011	China	Chinese	Liver Cancer	401/459	0.751	0.285	HB	SNP Stream
Xiong X D	2014	China	Chinese	Cervical Cancer	103/417	0.255	0.261	HB	DNA Sequencing
Nikolic Z	2015	Serbia	Serbian	Prostate cancer	353/308	0.101	0.284	HB	PCR–RFLP

Abbreviations: HWE, Hardy-Weinberg equilibrium; MAF, minor allele frequency; HB, Hospital based controls; PB, population based controls; PCR, Polymerase chain reaction; RFLP, Restriction fragment length polymorphism; MALDI-TOF, Matrix-assisted laser desorption/ ionization- time of flight.

### Quantitative synthesis

**Figure 2:**
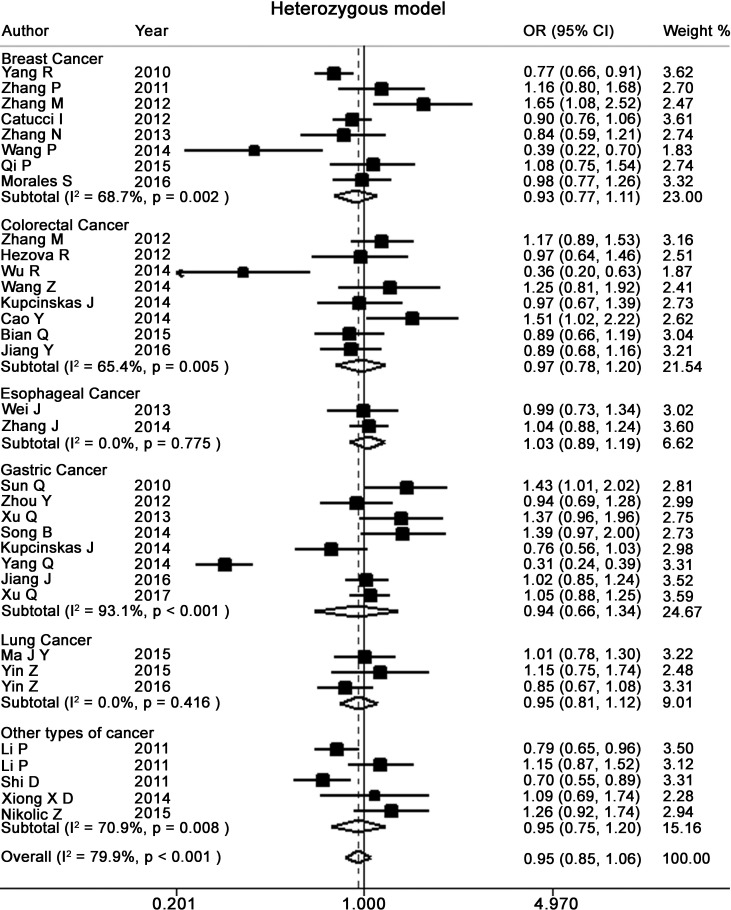
Forest plots of heterozygote for meta-analysis on the association of rs895819 with cancer risk The squares and horizontal lines correspond to OR and 95% CI of specific study, and the area of squares reflects study weight (inverse of the variance). The diamond represents the pooled OR and its 95% CI.

**Figure 3:**
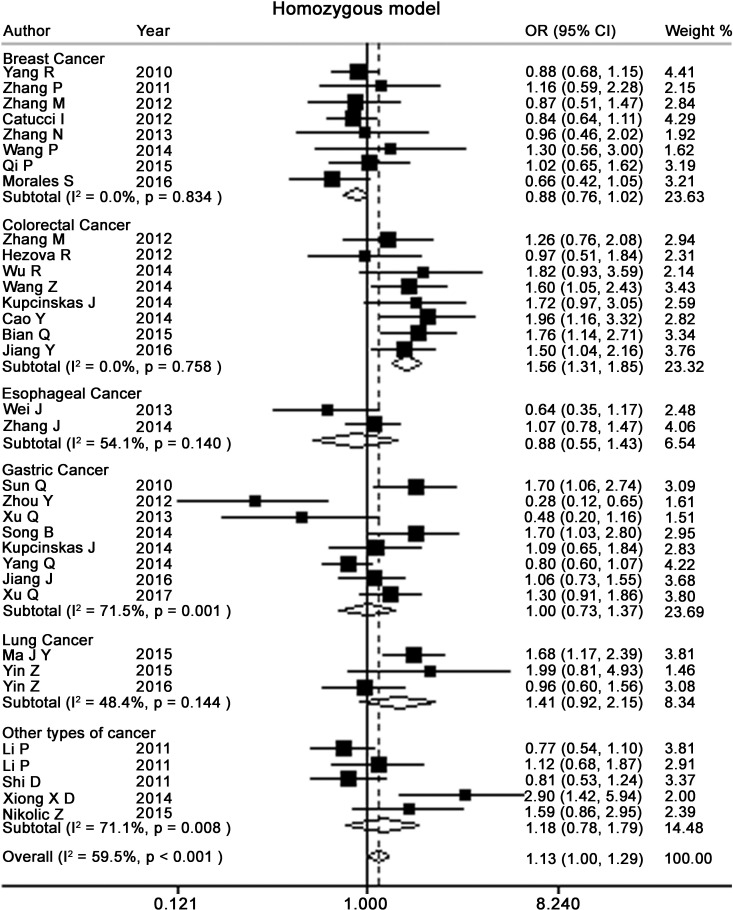
Forest plots of homozygote model for meta-analysis on the association of rs895819 with cancer risk The squares and horizontal lines correspond to OR and 95% CI of specific study, and the area of squares reflects study weight (inverse of the variance). The diamond represents the pooled OR and its 95% CI.

**Figure 4:**
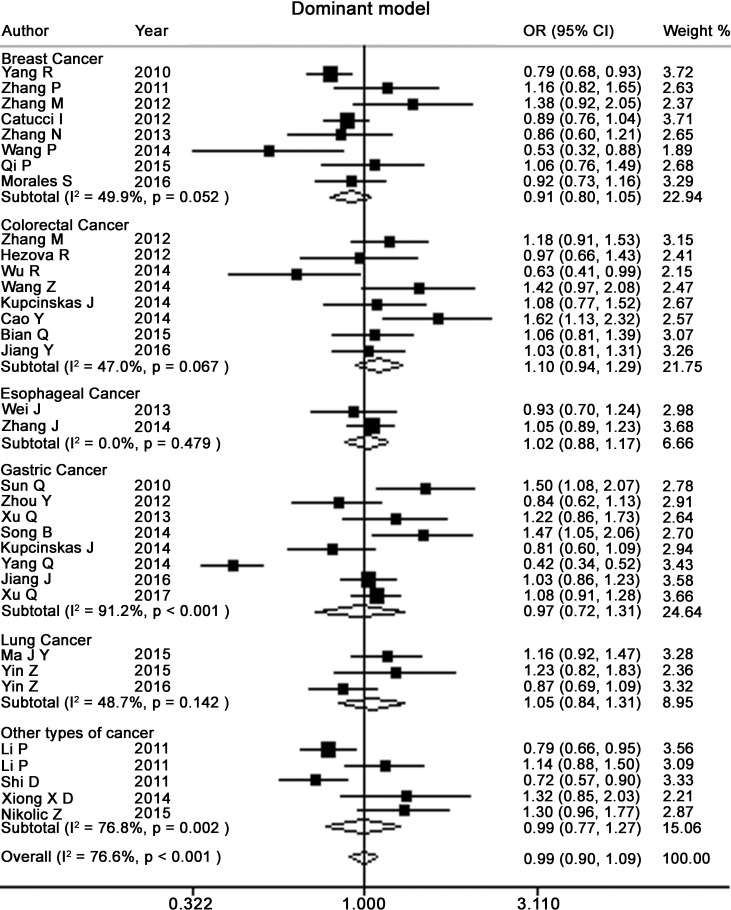
Forest plots of dominant model for meta-analysis on the association of rs895819 with cancer risk The squares and horizontal lines correspond to OR and 95% CI of specific study, and the area of squares reflects study weight (inverse of the variance). The diamond represents the pooled OR and its 95% CI.

**Figure 5:**
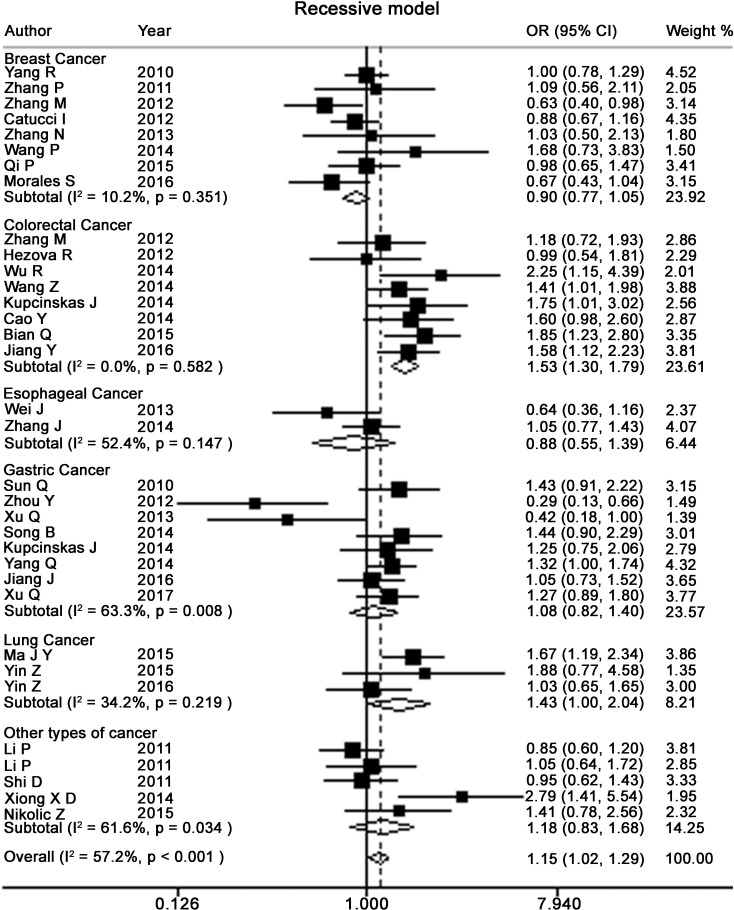
Forest plots of recessive model for meta-analysis on the association of rs895819 with cancer risk The squares and horizontal lines correspond to OR and 95% CI of specific study, and the area of squares reflects study weight (inverse of the variance). The diamond represents the pooled OR and its 95% CI.

**Figure 6:**
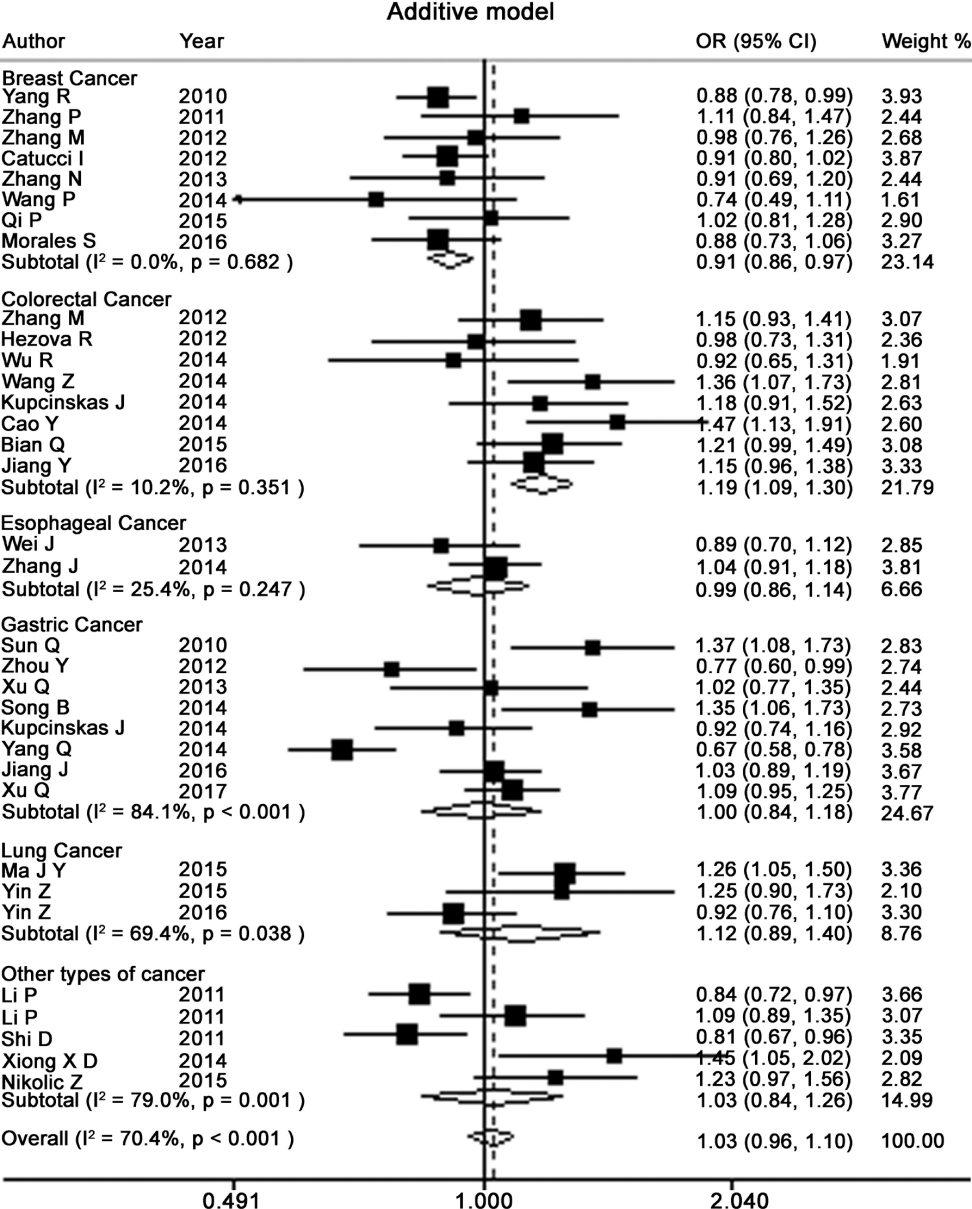
Forest plots of additive model for meta-analysis on the association of rs895819 with cancer risk The squares and horizontal lines correspond to OR and 95% CI of specific study, and the area of squares reflects study weight (inverse of the variance). The diamond represents the pooled OR and its 95% CI.

Table 2 presents the meta-analysis results for all cancers. By pooling all the studies, rs895819 was associated with increased risk of cancer in recessive (OR = 1.15; 95% CI = 1.02–1.29) but not other model (Figures 2–6). In the subgroup analyses, rs895819 was associated with increased risk of cancer in homogeneous (OR = 1.17; 95% CI = 1.01–1.36) or recessive (OR = 1.18; 95% CI = 1.03–1.35) model in Asians, but no association of rs895819 with cancer risk was found in Caucasians. (Table 2). Interestingly, the [G] allele of rs895819 was significantly associated with decreased risk of breast cancer (OR = 0.91; 95% CI = 0.86–0.97). However, rs895819 was associated with increased risk of colorectal cancer in homogeneous (OR = 1.56; 95% CI = 1.31–1.85), recessive (OR = 1.53; 95% CI = 1.30–1.79) or additive model (OR = 1.19; 95% CI = 1.09–1.30), and with increased risk of lung cancer in recessive model (OR = 1.43; 95% CI = 1.00–2.04). In addition, rs895819 was not associated with risk of esophageal cancer or gastric cancer. For other types of cancers, pooled analysis showed lack of association between rs895819 and cancer risk, and we did not perform a meta-analysis for each cancer since only one study was included for different type cancer. In stratified analysis by the sources of controls, the rs895819 was significantly associated with increased cancer risk in homogeneous (OR = 1.21; 95% CI = 1.03–1.42), or recessive (OR = 1.21; 95% CI = 1.04–1.41) model when pooling twenty-six hospital-based case-control studies, but no association was found when pooling eight population-based studies.

**Table 2:** Stratified analysis of the association between miR-27a polymorphisms and cancer risk

**Groups**	***n***^a^	**Heterogenous**		**Homogenous**		**Dominant**		**Recessive**		**Additive**	
		**OR (95% CI)**^b^	***P***^c^	**OR (95% CI)**^b^	***P***^c^	**OR (95% CI)**^b^	***P***^c^	**OR (95% CI)**^**b**^	***P***^c^	**OR (95% CI)**^b^	***P***^c^
All	34	0.95 (0.85–1.06)	< 0.001	1.13 (1.00–1.29)	< 0.001	0.99 (0.91–1.09)	< 0.001	**1.15 (1.02–1.29)**	< 0.001	1.03 (0.96–1.10)	< 0.001
Cancer types											
Breast cancer	8	0.93 (0.77–1.11)	0.002	0.88 (0.76–1.02)	0.834	0.91 (0.80–1.05)	0.052	0.90 (0.77–1.05)	0.351	**0.91 (0.86–0.97)**	0.682
Gastric cancer	8	0.94 (0.66–1.34)	< 0.001	1.00 (0.74–1.37)	0.001	0.97 (0.72–1.31)	< 0.001	1.08 (0.83–1.40)	0.008	1.00 (0.84–1.19)	< 0.001
Colorectal cancer	8	0.97 (0.78–1.20)	0.005	**1.56 (1.31–1.85)**	0.758	1.10 (0.94–1.29)	0.067	**1.53 (1.30–1.79)**	0.582	**1.19 (1.09–1.30)**	0.351
Lung cancer	3	0.95 (0.81–1.12)	0.416	1.41 (0.92–2.15)	0.144	1.05 (0.84–1.31)	0.142	**1.43 (1.00–2.04)**	0.219	1.12 (0.89–1.40)	0.038
Esophageal cancer	2	1.03 (0.89–1.19)	0.775	0.88 (0.55–1.43)	0.140	1.02 (0.88–1.17)	0.479	0.88 (0.55–1.40)	0.147	0.99 (0.86–1.14)	0.247
Other types	5	0.95 (0.75–1.20)	0.008	1.18 (0.78–1.79)	0.008	0.99 (0.77–1.27)	0.002	1.18 (0.83–1.68)	0.034	1.03 (0.84–1.26)	0.001
Ethnic											
Asian	27	0.96 (0.84–1.10)	< 0.001	**1.17 (1.01–1.36)**	< 0.001	1.01 (0.90–1.14)	< 0.001	**1.18 (1.03–1.35)**	< 0.001	1.04 (0.97–1.13)	< 0.001
Caucasian	7	0.91 (0.80–1.02)	0.146	0.98 (0.79–1.22)	0.110	0.92 (0.82–1.03)	0.131	1.03 (0.84–1.26)	0.124	0.96 (0.87–1.05)	0.114
Source of controls											
HB	26	0.97 (0.89–1.07)	< 0.001	**1.21 (1.03–1.42)**	< 0.001	1.02 (0.93–1.12)	< 0.001	**1.21 (1.04–1.41)**	< 0.001	1.06 (0.98–1.14)	< 0.001
PB	8	0.91 (0.67–1.24)	< 0.001	0.92 (0.81–1.05)	0.736	0.92 (0.72–1.18)	< 0.001	1.01 (0.87–1.16)	0.222	0.94 (0.84–1.06)	< 0.001

^a^Number of comparisons.

^b^The crude OR and 95% CI were calculated based on the genotype frequencies.

^c^*P* value of *Q*-test for heterogeneity analysis.

Next, we performed subgroup analyses for specific type of cancer. As to breast cancer, except for recessive model, rs895819 is associated with reduced cancer risk in Caucasians but not Asians (Supplementary Table 1). For cancers from digestive system, we found significant association between rs895819 and increased risk of cancers from all digestive system when pooling 19 studies on esophageal, gastric, colorectal and liver cancers in recessive (OR = 1.23; 95% CI = 1.06–1.43) or homogeneous model (OR = 1.20; 95% CI = 1.01–1.43). This association remained in digestive tracts when pooling 18 studies on esophageal, gastric and colorectal cancers in recessive (OR = 1.24; 95% CI, 1.06–1.45) or homogeneous model (OR = 1.20; 95% CI = 1.00–1.44). However, no association was found between rs895819 and risk of upper aero digestive tract cancers when pooling 10 studies on esophageal and gastric cancers. For gastric cancer, rs895819 was not associated with risk of gastric cancer in Asians when pooling 7 studies. For colorectal cancer, we observed that rs895819 were associated with increased risk of colorectal cancer in Asians in homogenous, recessive or additive model, but no association was found in Caucasians.

### Sensitivity analysis and publication bias

When pooling all eligible studies, sensitivity analysis showed that exclusion of each study did not influence the result in specific genotype comparison for rs895819 except dominant model, suggesting that the results of synthetic analysis were robust for other each model (Supplementary Figure 1).

**Figure 7:**
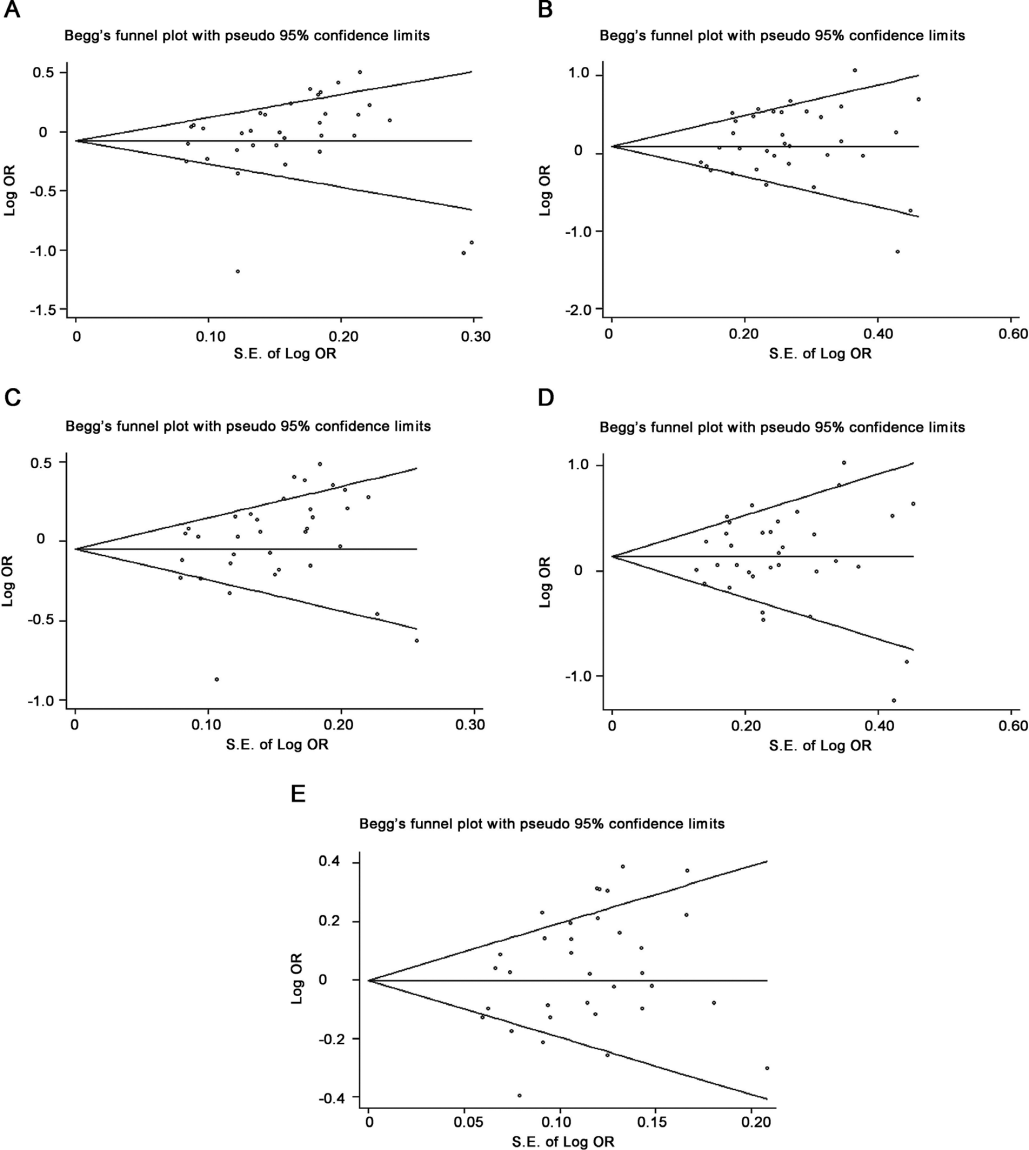
Funnel plots showed symmetric or asymmetric distribution Log OR was plotted against the standard error of log OR for studies on rs895819 in heterozygous (**A**), homozygous (**B**), dominant (**C**), recessive (**D**) or additive model (**E**). The dots represent specific studies for the indicated association.

The Begg’s test showed that the *P* value of rs895819 was 0.173, 0.553, 0.097, 0.767 or 0.192 for heterozygous, homozygous, dominant recessive and additive model, respectively, while the corresponding funnel plots showed symmetric distribution ( Figure 7). The Egger’s test also showed that all the *P* values of rs895819 was 0.405, 0.293, 0.085, 0.941 or 0.053 for heterozygous, homozygous, dominant recessive and additive model, respectively, suggesting that there was no significant publication bias in the present study.

## DISCUSSION

In this study, we performed an update meta-analysis and found that rs895819 was associated with increased cancer risk in recessive model when including 34 studies of all cancers (15,388 cases and 18,704 controls), and this association remained in Asians but not Caucasians. Interestingly, the [G] allele of rs895819 played protective role on breast cancer, but the rs895819 was associated with increased risk of colorectal cancer or lung cancer in recessive model. In addition, no association was found between rs895819 and risk of gastric cancer or esophageal cancer.

Based on 17 case-control studies with 7,813 cases and 9,602 controls, our previous meta-analysis did not suggest any association between rs895819 and cancer susceptibility, while rs895819 was associated with a reduced cancer risk in heterozygous, dominant or additive model in Caucasians but not in Asians [33]. By pooling 19 studies (17 articles) involving 7,800 cases and 9,060 controls, a recent study by Feng et al., failed to find any associations between rs895819 polymorphism and cancer risk, while statistically significantly reduced cancer risks were found among Asians for dominant contrast and a subtly decreased risk was observed in the Caucasian population for heterozygous or additive contrast [29]. In the present study, by including 34 studies with almost twice number of subjects, we found that rs895819 was associated with increased cancer risk in recessive model for all population and Asians, but not Caucasians, suggesting a possible ethnic difference in the genetic and the environmental factors. The discrepancy between these meta-analyses might be due to sample size of pooled studies, and whether the risk of rs895819 on cancer depends on ethnicity should be confirmed by more studies.

When stratified by the cancer type, our data was consistent with previous meta-analysis reports that the [G] allele of rs895819 was associated with decreased risk in breast cancer for all population and Caucasians, but not Asians [29, 33]. For colorectal cancer, the study by Liu et al., pooling seven studies with 2,230 cases and 2,775 controls provided a moderate evidence for the association between rs895819 and increased risk of colorectal cancer under multiple genetic models for all population and Asians, but not Caucasians [28]. By including one more study, our data showed consistent findings. However, no significant association was found in cancers from upper aero digestive tracts, stomach or esophagus. As to lung cancer, we for the first time showed an association of rs895819 with increased risk of lung cancer in dominant model although the included studies were very limited. These findings suggested distinct effects of rs895819 on carcinogenesis in different types of cancers.

Generally, miR-27a, as an onco-miR, exhibits its oncogenic activity through dysregulating its downstream targets, and plays critical roles in the pathogenesis of multiple cancer types, e.g., cancer cell clonogenic growth and metastatic abilities [68–70]. Although the binding of the mature miRNA to target mRNAs may not be influenced by the rs895819 since rs895819 is not located in seeding sites [26], polymorphisms in the loop of pre-miRNAs could influence mature miRNAs processing and the expression levels of their mature forms [71]. Previous studies showed that rs895819 was positive associated with serum expression of mature miR-27a in gastric cancer patients [23, 24], but the molecular mechanism on regulation of miR-27a expression by rs895819 has not been investigated. It remains unclear whether rs895819 affected the processing of miR-27a maturation or/and expression of mature miR-27a. Our study showed distinct effects of rs895819 on cancer risk in different types of cancers, e.g., reduced risk of breast cancer *vs*. increased risk of colorectal or lung cancer, suggesting various roles of rs895819 in different cancer development since pre-miRNA is processed into mature miRNA via complex mechanisms.

The major limitation of this study is the heterogeneity for the rs895819 among these studies on different ethnic populations, even with same type cancer, and different types of cancers. The heterogeneity may come from various factors, such as diversity in characteristics of subjects, differences in the study population and study design, genetic susceptibility to different cancers, and different genotyping strategies. To eliminate heterogeneity, we performed subgroup analyses with a random-effects model to pool the studies when the significant heterogeneity was present. Secondly, we pooled the data based on unadjusted information without of considering the combination genetic factors together with environmental exposures due to lack of individual data for a more precise analysis. Thirdly, in some subgroup analyses, e.g., lung cancer, limited studies included may lead to reduced statistical power. Fourthly, sensitivity analysis showed that exclusion of one of few studies influenced the result in dominant model of genotype comparison for rs895819. This may be due to boardline significance of the association. Fifthly, our findings on the association of rs895819 with risk of specific cancer were mathematically significant, but the real effects of rs895819 on specific cancer risk in real SNP model await further investigations. Finally, our Egger’s and Begg’s test showed that slight publication bias exists, because only published studies in English or Chinese were included in this meta-analysis, which might affect the results.

In summary, current data suggest that rs895819 may contribute to increased susceptibility to colorectal and lung cancers, but appears as a protective factor for breast cancer. Since the studies on specific cancer included in this meta-analysis were still limited, the explanation of the current findings should be with caution and further well-designed studies with larger populations are required to clarify the distinct effects of rs895819 on cancer development in different types of cancers.

## MATERIALS AND METHODS

### Identification and eligibility of relevant studies

To identify the studies on the relationship between *miR-27a* polymorphism and cancer risk, we conducted systemic literature searching by retrieving databases and manual searching. Firstly, the PubMed databases up to February 2017 were searched using the following keywords: “miR-27a”, “polymorphism” and “cancer”. Additional manual searches were performed from other databases, e.g., Web of Science, China National Knowledge Infrastructure (CNKI), and references of review articles or original studies on this topic. The eligible studies met the following criteria: (a) case-control study, (b) available genotype frequency for the SNP investigated, and (c) sufficient data to estimate an odds ratio (OR) with corresponding 95% confidence interval (CI).

### Data extraction

Two investigators (M.C and W.F.) independently reviewed the studies included, extracted data and reached a consensus on all of the items if discrepancy existed. The following information of each study was extracted: first author and year of article, country of origin, ethnicity of subjects, cancer types, number of cases and controls, Hardy-Weinberg equilibrium (HWE) test for the genotype frequency of controls, minor allele frequencies (MAF) of the controls, source of controls and genotyping method. The ethnicity descents were categorized as Asian and Caucasian, and the cancer types were grouped as breast cancer, colorectal cancer, gastric cancer, lung cancer, esophageal cancer and others, and the sources of controls were defined as population-based (HB) or hospital-based (PB) respectively.

### Statistical analysis

The Hardy-Weinberg equilibrium (HWE) was tested by the chi-square goodness of fit test. The crude ORs with 95% CIs were used to assess the strength of association between the *miR-27a* polymorphism rs895819 and cancer risk. Firstly, the risks of the AG and GG genotypes on cancer were estimated when comparing with the reference AA homozygote. Secondly, the risks of (AG + GG *vs*. AA) and (GG *vs*. AA + AG) on cancer were evaluated, assuming dominant and recessive effects of the variant GG allele, respectively. Thirdly, the effect of [G] allele on cancer risk were examined by comparing with the reference [A] allele (additive model). Stratified analyses were conducted by ethnicities of subjects, types of cancer and sources of controls. For the specific cancer, subgroup analyses were performed by ethnicity as well. Potential heterogeneity was evaluated by the I^2^-based *Q*-test. A random-effects (DerSimonian-Laird method) was used to calculate pooled effect estimates. Both Egger’s test [30] and Begg’s test [31] were applied to examine the publication bias for the overall pooled analyses of rs895819. In addition, Begg’s funnel plots were drawn and the asymmetries of the funnel plots were applied to evaluate potential publication bias. For the one-way sensitivity analysis, each study was excluded each time, and the new pooled results reflected the influence of the deleted study to the overall summary OR. All analyses were carried out with Stata software (StataCorp LP, College Station, TX), and the statistical tests were considered statistically significant at *P* value < 0.05 (two-sided).

## SUPPLEMENTARY MATERIALS FIGURE AND TABLE



## References

[1] Iwakawa HO, Tomari Y (2015). The Functions of MicroRNAs: mRNA Decay and Translational Repression. Trends Cell Biol.

[2] Bartel DP (2009). MicroRNAs: target recognition and regulatory functions. Cell.

[3] Mendell JT, Olson EN (2012). MicroRNAs in stress signaling and human disease. Cell.

[4] Chen K, Rajewsky N (2006). Natural selection on human microRNA binding sites inferred from SNP data. Nat Genet.

[5] Saunders MA, Liang H, Li WH (2007). Human polymorphism at microRNAs and microRNA target sites. Proc Natl Acad Sci U S A.

[6] Ryan BM, Robles AI, Harris CC (2010). Genetic variation in microRNA networks: the implications for cancer research. Nat Rev Cancer.

[7] Chen K, Song F, Calin GA, Wei Q, Hao X, Zhang W (2008). Polymorphisms in microRNA targets: a gold mine for molecular epidemiology. Carcinogenesis.

[8] Jiang J, Lv X, Fan L, Huang G, Zhan Y, Wang M, Lu H (2014). MicroRNA-27b suppresses growth and invasion of NSCLC cells by targeting Sp1. Tumour biology : the journal of the International Society for Oncodevelopmental Biology and Medicine.

[9] Tanaka K, Miyata H, Sugimura K, Fukuda S, Kanemura T, Yamashita K, Miyazaki Y, Takahashi T, Kurokawa Y, Yamasaki M, Wada H, Nakajima K, Takiguchi S, Mori M, Doki Y (2015). miR-27 is associated with chemoresistance in esophageal cancer through transformation of normal fibroblasts to cancer-associated fibroblasts. Carcinogenesis.

[10] Tang W, Zhu J, Su S, Wu W, Liu Q, Su F, Yu F (2012). MiR-27 as a prognostic marker for breast cancer progression and patient survival. PloS one.

[11] Zhang Z, Liu S, Shi R, Zhao G (2011). miR-27 promotes human gastric cancer cell metastasis by inducing epithelial-to-mesenchymal transition. Cancer genetics.

[12] Zhou L, Liang X, Zhang L, Yang L, Nagao N, Wu H, Liu C, Lin S, Cai G, Liu J (2016). MiR-27a-3p functions as an oncogene in gastric cancer by targeting BTG2. Oncotarget.

[13] Catucci I, Verderio P, Pizzamiglio S, Bernard L, Dall'olio V, Sardella D, Ravagnani F, Galastri L, Barile M, Peissel B, Zaffaroni D, Manoukian S, Radice P, Peterlongo P (2012). The SNP rs895819 in miR-27a is not associated with familial breast cancer risk in Italians. Breast cancer research and treatment.

[14] Morales S, Gulppi F, Gonzalez-Hormazabal P, Fernandez-Ramires R, Bravo T, Reyes JM, Gomez F, Waugh E, Jara L (2016). Association of single nucleotide polymorphisms in Pre-miR-27a, Pre-miR-196a2, Pre-miR-423, miR-608 and Pre-miR-618 with breast cancer susceptibility in a South American population. BMC genetics.

[15] Qi P, Wang L, Zhou B, Yao WJ, Xu S, Zhou Y, Xie ZB (2015). Associations of miRNA polymorphisms and expression levels with breast cancer risk in the Chinese population. Genetics and molecular research : GMR.

[16] Wang P (2014). The role of microRNA in breast cancer prevention and diagnosis.

[17] Yang R, Schlehe B, Hemminki K, Sutter C, Bugert P, Wappenschmidt B, Volkmann J, Varon R, Weber BH, Niederacher D, Arnold N, Meindl A, Bartram CR, Schmutzler RK, Burwinkel B (2010). A genetic variant in the pre-miR-27a oncogene is associated with a reduced familial breast cancer risk. Breast cancer research and treatment.

[18] Zhang M, Jin M, Yu Y, Zhang S, Wu Y, Liu H, Liu H, Chen B, Li Q, Ma X, Chen K (2012). Associations of miRNA polymorphisms and female physiological characteristics with breast cancer risk in Chinese population. European journal of cancer care.

[19] Zhang N, Huo Q, Wang X, Chen X, Long L, Jiang L, Ma T, Yang Q (2013). A genetic variant in pre-miR-27a is associated with a reduced breast cancer risk in younger Chinese population. Gene.

[20] Zhang P

[21] Jiang J, Jia ZF, Cao DH, Wu YH, Sun ZW, Cao XY (2016). Association of the miR-146a rs2910164 polymorphism with gastric cancer susceptibility and prognosis. Future oncology.

[22] Kupcinskas J, Wex T, Link A, Leja M, Bruzaite I, Steponaitiene R, Juzenas S, Gyvyte U, Ivanauskas A, Ancans G, Petrenkiene V, Skieceviciene J, Kupcinskas L, Malfertheiner P (2014). Gene polymorphisms of micrornas in Helicobacter pylori-induced high risk atrophic gastritis and gastric cancer. PloS one.

[23] Song B, Yan G, Hao H, Yang B (2014). rs11671784 G/A and rs895819 A/G polymorphisms inversely affect gastric cancer susceptibility and miR-27a expression in a Chinese population. Medical science monitor.

[24] Sun Q, Gu H, Zeng Y, Xia Y, Wang Y, Jing Y, Yang L, Wang B (2010). Hsa-mir-27a genetic variant contributes to gastric cancer susceptibility through affecting miR-27a and target gene expression. Cancer science.

[25] Xu Q, He CY, Liu JW, Yuan Y (2013). Pre-miR-27a rs895819A/G polymorphisms in cancer: a meta-analysis. PloS one.

[26] Yang Q, Jie Z, Ye S, Li Z, Han Z, Wu J, Yang C, Jiang Y (2014). Genetic variations in miR-27a gene decrease mature miR-27a level and reduce gastric cancer susceptibility. Oncogene.

[27] Zhou Y, Du WD, Chen G, Ruan J, Xu S, Zhou FS, Zuo XB, Lv ZJ, Zhang XJ (2012). Association analysis of genetic variants in microRNA networks and gastric cancer risk in a Chinese Han population. Journal of cancer research and clinical oncology.

[28] Liu F, Dear K, Huang L, Liu L, Shi Y, Nie S, Liu Y, Lu Y, Xiang H (2016). Association between microRNA-27a rs895819 polymorphism and risk of colorectal cancer: A meta-analysis. Cancer genetics.

[29] Feng Y, Duan F, Song C, Zhao X, Dai L, Cui S (2016). Systematic evaluation of cancer risk associated with rs2292832 in miR149 and rs895819 in miR27a: a comprehensive and updated metaanalysis. Oncotarget.

[30] Hayashino Y, Noguchi Y, Fukui T (2005). Systematic evaluation and comparison of statistical tests for publication bias. J Epidemiol.

[31] Begg CB, Mazumdar M (1994). Operating characteristics of a rank correlation test for publication bias. Biometrics.

[32] Yang R, Burwinkel B (2012). A bias in genotyping the miR-27a rs895819 and rs11671784 variants. Breast cancer research and treatment.

[33] Bai RP, Weng Y, Su LL, Jin MJ, Xu ZP, Lu LQ, Chen GD (2014). Association of a pre-miR-27a polymorphism with cancer risk: an updated meta-analysis. Asian Pacific journal of cancer prevention : APJCP.

[34] Dai ZJ, Shao YP, Wang XJ, Xu D, Kang HF, Ren HT, Min WL, Lin S, Wang M, Song ZJ (2015). Five common functional polymorphisms in microRNAs (rs2910164, rs2292832, rs11614913, rs3746444, rs895819) and the susceptibility to breast cancer: evidence from 8361 cancer cases and 8504 controls. Current pharmaceutical design.

[35] Hu Y, Yu CY, Wang JL, Guan J, Chen HY, Fang JY (2014). MicroRNA sequence polymorphisms and the risk of different types of cancer. Scientific reports.

[36] Ma XP, Zhang T, Peng B, Yu L, Jiang de K (2013). Association between microRNA polymorphisms and cancer risk based on the findings of 66 case-control studies. PloS one.

[37] Wang Z, Lai J, Wang Y, Nie W, Guan X (2013). The Hsa-miR-27a rs895819 (A>G) polymorphism and cancer susceptibility. Gene.

[38] Chen QH, Wang QB, Zhang B (2014). Ethnicity modifies the association between functional microRNA polymorphisms and breast cancer risk: a HuGE meta-analysis. Tumour biology : the journal of the International Society for Oncodevelopmental Biology and Medicine.

[39] Wang B, Ma N, Wang Y (2012). Association between the hsa-mir-27a variant and breast cancer risk: a meta-analysis. Asian Pacific journal of cancer prevention : APJCP.

[40] Yuan L, Zhang TT, Ren Y (2015). miR-27a rs895819 polymorphism and risk of cancer in Chinese population: a meta-analysis. Journal of evidence-based medicine.

[41] Zhong S, Chen Z, Xu J, Li W, Zhao J (2013). Pre-mir-27a rs895819 polymorphism and cancer risk: a meta-analysis. Molecular biology reports.

[42] Xu Q, Liu JW, Yuan Y (2015). Comprehensive assessment of the association between miRNA polymorphisms and gastric cancer risk. Mutation research Reviews in mutation research.

[43] Jahid S, Sun J, Edwards RA, Dizon D, Panarelli NC, Milsom JW, Sikandar SS, Gumus ZH, Lipkin SM (2012). miR-23a promotes the transition from indolent to invasive colorectal cancer. Cancer discovery.

[44] Hirota T, Date Y, Nishibatake Y, Takane H, Fukuoka Y, Taniguchi Y, Burioka N, Shimizu E, Nakamura H, Otsubo K, Ieiri I (2012). Dihydropyrimidine dehydrogenase (DPD) expression is negatively regulated by certain microRNAs in human lung tissues. Lung cancer.

[45] Deng Y, Bai H, Hu H (2015). rs11671784 G/A variation in miR-27a decreases chemo-sensitivity of bladder cancer by decreasing miR-27a and increasing the target RUNX-1 expression. Biochemical and biophysical research communications.

[46] Falvella FS, Cheli S, Martinetti A, Mazzali C, Iacovelli R, Maggi C, Gariboldi M, Pierotti MA, Di Bartolomeo M, Sottotetti E, Mennitto R, Bossi I, de Braud F, Clementi E, Pietrantonio F (2015). DPD and UGT1A1 deficiency in colorectal cancer patients receiving triplet chemotherapy with fluoropyrimidines, oxaliplatin and irinotecan. British journal of clinical pharmacology.

[47] Rah H, Kim HS, Cha SH, Kim YR, Lee WS, Ko JJ, Kim NK (2015). Association of breast cancer-related microRNA polymorphisms with idiopathic primary ovarian insufficiency. Menopause.

[48] Fang C, Li XP, Gong WJ, Wu NY, Tang J, Yin JY, Li X, Zhang W, Zhou HH, Liu ZQ (2016). Age-related common miRNA polymorphism associated with severe toxicity in lung cancer patients treated with platinum-based chemotherapy. Clinical and experimental pharmacology & physiology.

[49] Xu J, Yin Z, Shen H, Gao W, Qian Y, Pei D, Liu L, Shu Y (2013). A genetic polymorphism in pre-miR-27a confers clinical outcome of non-small cell lung cancer in a Chinese population. PloS one.

[50] Song MY, Su HJ, Zhang L, Ma JL, Li JY, Pan KF, You WC (2013). Genetic polymorphisms of miR-146a and miR-27a, H. pylori infection, and risk of gastric lesions in a Chinese population. PloS one.

[51] Gupta A, Sharma A, Yadav A, Rastogi N, Agrawal S, Kumar A, Kumar V, Misra S, Mittal B (2015). Evaluation of miR-27a, miR-181a, and miR-570 genetic variants with gallbladder cancer susceptibility and treatment outcome in a North Indian population. Molecular diagnosis & therapy.

[52] Meulendijks D, Henricks LM, Amstutz U, Froehlich TK, Largiader CR, Beijnen JH, de Boer A, Deenen MJ, Cats A, Schellens JH (2016). Rs895819 in MIR27A improves the predictive value of DPYD variants to identify patients at risk of severe fluoropyrimidine-associated toxicity. International journal of cancer.

[53] Amstutz U, Offer SM, Sistonen J, Joerger M, Diasio RB, Largiader CR (2015). Polymorphisms in MIR27A Associated with Early-Onset Toxicity in Fluoropyrimidine-Based Chemotherapy. Clinical cancer research.

[54] Zhang J, Huang X, Xiao J, Yang Y, Zhou Y, Wang X, Liu Q, Yang J, Wang M, Qiu L, Zheng Y, Zhang P, Li J, Wang Y, Wei Q, Jin L (2014). Pri-miR-124 rs531564 and pri-miR-34b/c rs4938723 polymorphisms are associated with decreased risk of esophageal squamous cell carcinoma in Chinese populations. PloS one.

[55] Stenholm L, Stoehlmacher-Williams J, Al-Batran SE, Heussen N, Akin S, Pauligk C, Lehmann S, Senff T, Hofheinz RD, Ehninger G, Kramer M, Goekkurt E (2013). Prognostic role of microRNA polymorphisms in advanced gastric cancer: a translational study of the Arbeitsgemeinschaft Internistische Onkologie (AIO). Annals of oncology.

[56] Zanetti KA, Haznadar M, Welsh JA, Robles AI, Ryan BM, McClary AC, Bowman ED, Goodman JE, Bernig T, Chanock SJ, Harris CC (2012). 3'-UTR and functional secretor haplotypes in mannose-binding lectin 2 are associated with increased colon cancer risk in African Americans. Cancer research.

[57] Wang Y, He Y, Qin Z, Jiang Y, Jin G, Ma H, Dai J, Chen J, Hu Z, Guan X, Shen H (2014). Evaluation of functional genetic variants at 6q25.1 and risk of breast cancer in a Chinese population. Breast cancer research: BCR.

[58] He F, Lin J, Yu T, Zhang X, Liu Z, Xiong W, Cai L (2016). [Interaction research on smoking and microRNA genes SNP related to lung cancer in Fujian Han population]. Zhonghua yu fang yi xue za zhi [Chinese journal of preventive medicine].

[59] Yuan L, Chu H, Wang M, Gu X, Shi D, Ma L, Zhong D, Du M, Li P, Tong N, Fu G, Qin C, Yin C, Zhang Z (2013). Genetic variation in DROSHA 3'UTR regulated by hsa-miR-27b is associated with bladder cancer risk. PloS one.

[60] Yoon KA, Yoon H, Park S, Jang HJ, Zo JI, Lee HS, Lee JS (2012). The prognostic impact of microRNA sequence polymorphisms on the recurrence of patients with completely resected non-small cell lung cancer. The Journal of thoracic and cardiovascular surgery.

[61] Li PY (2011). Genetic associations of miRNA-SNPs with the common diseases and integrative analysis of “OMICS” data of hepatocellular carcinoma. Military Medical Sciences.

[62] Wu R (2014). The Association of miR-SNP with the Susceptibility of Colorectal Cancer and Response to Chemotherapy.

[63] Zhang MW

[64] Shi D, Li P, Ma L, Zhong D, Chu H, Yan F, Lv Q, Qin C, Wang W, Wang M, Tong N, Zhang Z, Yin C (2012). A genetic variant in pre-miR-27a is associated with a reduced renal cell cancer risk in a Chinese population. PLoS One.

[65] Xiong XD, Luo XP, Cheng J, Liu X, Li EM, Zeng LQ (2014). A genetic variant in pre-miR-27a is associated with a reduced cervical cancer risk in southern Chinese women. Gynecologic oncology.

[66] Nikolic Z, Savic Pavicevic D, Vucic N, Cidilko S, Filipovic N, Cerovic S, Vukotic V, Romac S, Brajuskovic G (2015). Assessment of association between genetic variants in microRNA genes hsa-miR-499, hsa-miR-196a2 and hsa-miR-27a and prostate cancer risk in Serbian population. Experimental and molecular pathology.

[67] Ma JY, Yan HJ, Yang ZH, Gu W (2015). Rs895819 within miR-27a might be involved in development of non small cell lung cancer in the Chinese Han population. Asian Pacific journal of cancer prevention. APJCP.

[68] Li WQ, Yu HY, Zhong NZ, Hou LJ, Li YM, He J, Liu HM, Xia CY, Lu YC (2015). miR27a suppresses the clonogenic growth and migration of human glioblastoma multiforme cells by targeting BTG2. International journal of oncology.

[69] Salah Z, Arafeh R, Maximov V, Galasso M, Khawaled S, Abou-Sharieha S, Volinia S, Jones KB, Croce CM, Aqeilan RI (2015). miR-27a and miR-27a* contribute to metastatic properties of osteosarcoma cells. Oncotarget.

[70] Zhang S, Ma C, Pang H, Zeng F, Cheng L, Fang B, Ma J, Shi Y, Hong H, Chen J, Wang Z, Xia J (2016). Arsenic trioxide suppresses cell growth and migration via inhibition of miR-27a in breast cancer cells. Biochemical and biophysical research communications.

[71] Zeng Y, Yi R, Cullen BR (2005). Recognition and cleavage of primary microRNA precursors by the nuclear processing enzyme Drosha. EMBO J.

