# Supplementary material for: Distinct effects of rs895819 on risk of different cancers: an update meta-analysis
Source: Oncotarget. 2017 Apr 27;8(43):75336–49. doi: 10.18632/oncotarget.17454 (PMC5650424; doi:10.18632/oncotarget.17454)
Supplement: Supplementary file 1 [file oncotarget-08-75336-s001.pdf]

Distinct effects of rs895819 on risk of different cancers: an update meta-analysis

Supplementary Materials

A

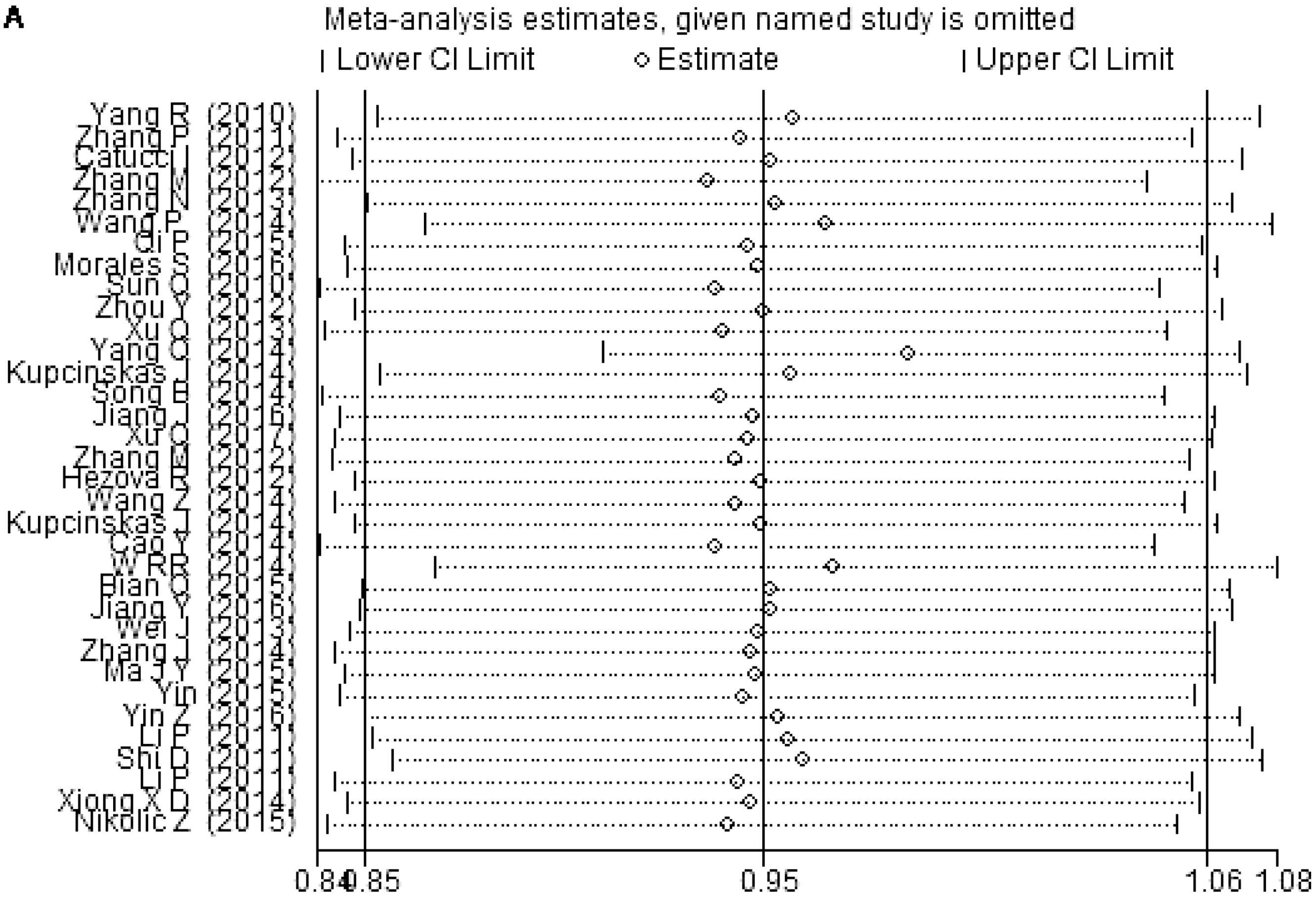

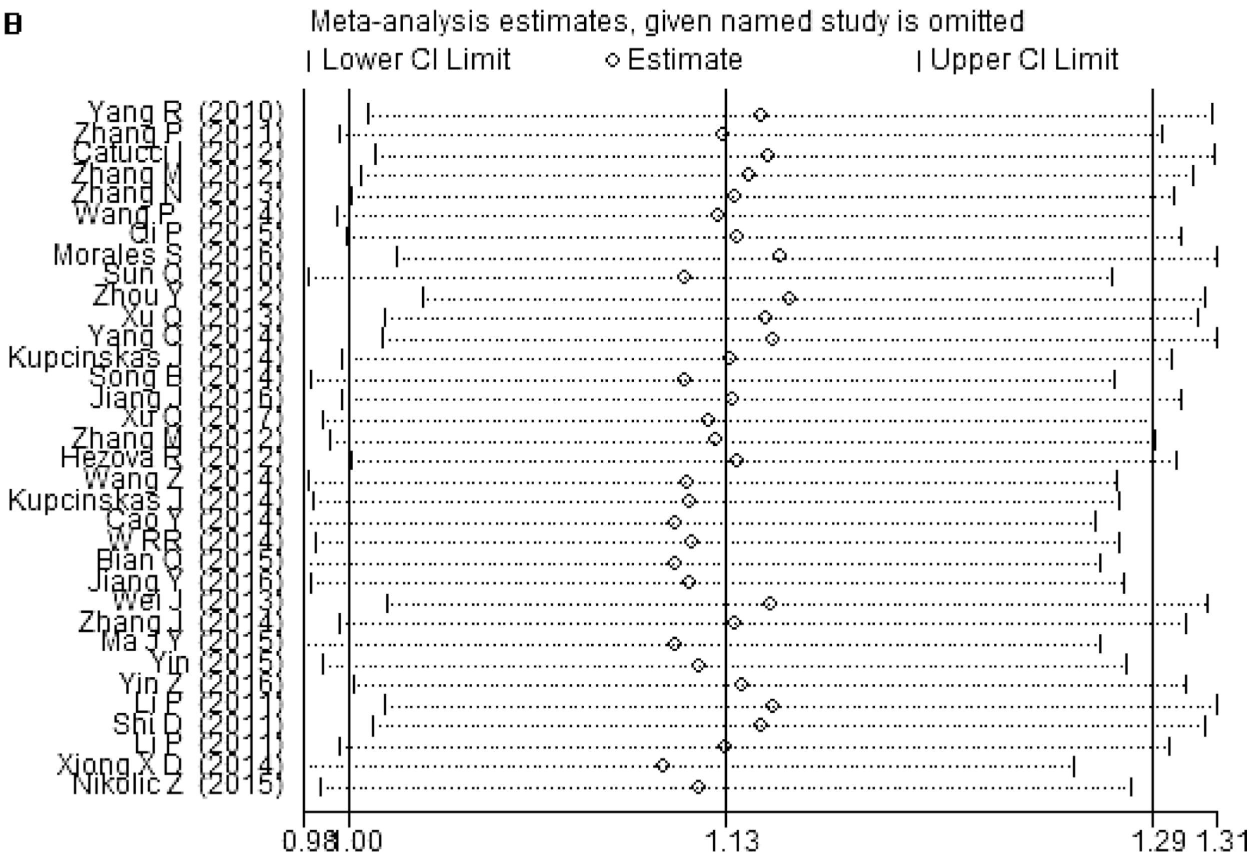
B
Meta-analysis estimates, given named study is omitted
Lower CI Limit
Estimate
Upper CI Limit
Yang R (2010)
Zhang P (2011)
Catucci I (2012)
Zhang M (2012)
Zhang N (2013)
Wang P (2014)
Qi P (2015)
Morales S (2016)
Sun Q (2010)
Zhou Y (2012)
Xu Q (2013)
Yang Q (2014)
Kupcinskas J (2014)
Song B (2014)
Jiang J (2016)
Xu Q (2017)
Zhang M (2012)
Hezova R (2012)
Wang Z (2014)
Kupcinskas J (2014)
Cao Y (2014)
W RR (2014)
Bian Q (2015)
Jiang Y (2016)
Wei J (2013)
Zhang J (2014)
Ma J Y (2015)
Yin (2015)
Yin Z (2016)
Li P (2011)
Shi D (2011)
Li P (2011)
Xiong X D (2014)
Nikolic Z (2015)
0.98
1.00
1.13
1.29
1.31

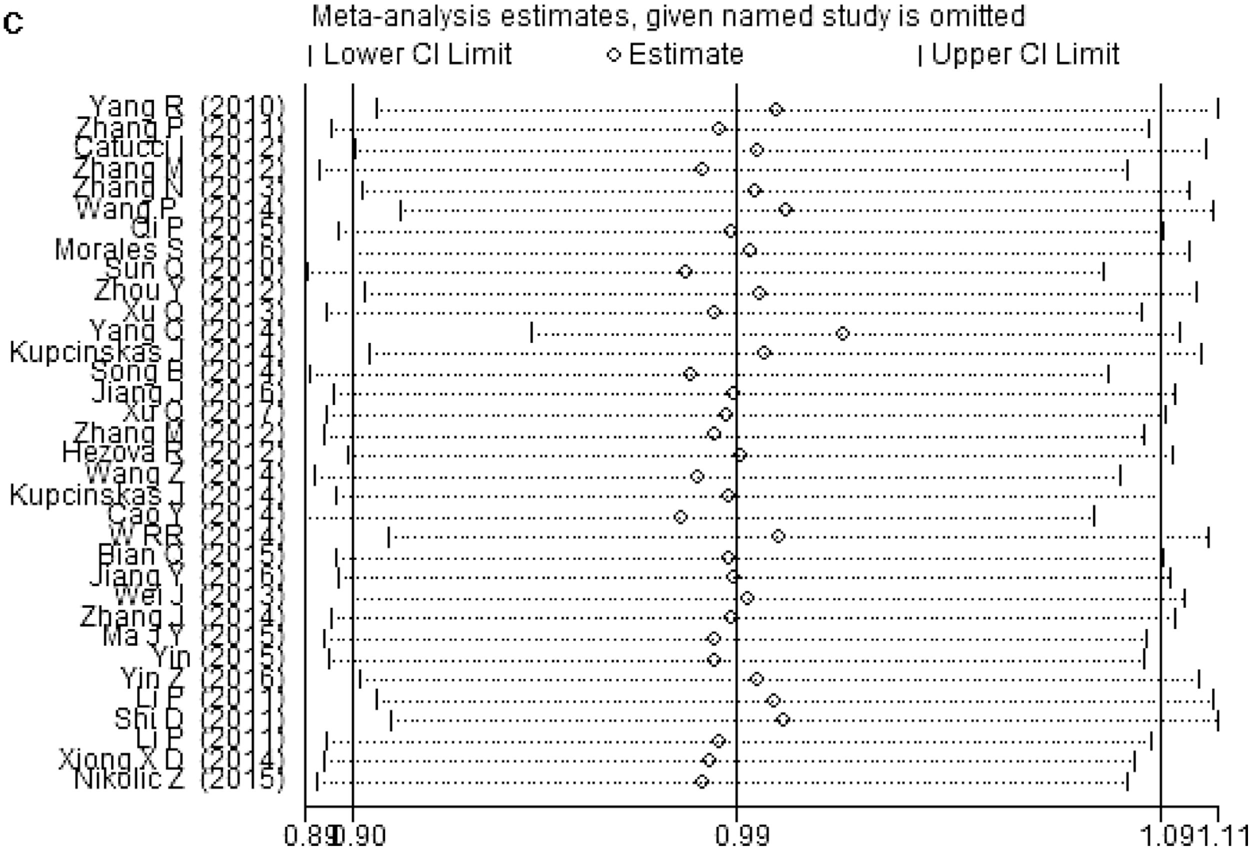
C
Meta-analysis estimates, given named study is omitted
Lower CI Limit
Estimate
Upper CI Limit
Yang R (2010)
Zhang P (2011)
Catucci I (2012)
Zhang M (2012)
Zhang N (2013)
Wang P (2014)
Qi P (2015)
Morales S (2016)
Sun Q (2010)
Zhou Y (2012)
Xu Q (2013)
Yang Q (2014)
Kupcinskas J (2014)
Song B (2014)
Jiang J (2016)
Xu Q (2017)
Zhang M (2012)
Hezova R (2012)
Wang Z (2014)
Kupcinskas J (2014)
Cao Y (2014)
W RR (2014)
Bian Q (2015)
Jiang Y (2016)
Wei J (2013)
Zhang J (2014)
Ma J Y (2015)
Yin (2015)
Yin Z (2016)
Li P (2011)
Shi D (2011)
Li P (2011)
Xiong X D (2014)
Nikolic Z (2015)
0.89
0.90
0.99
1.09
1.11

D

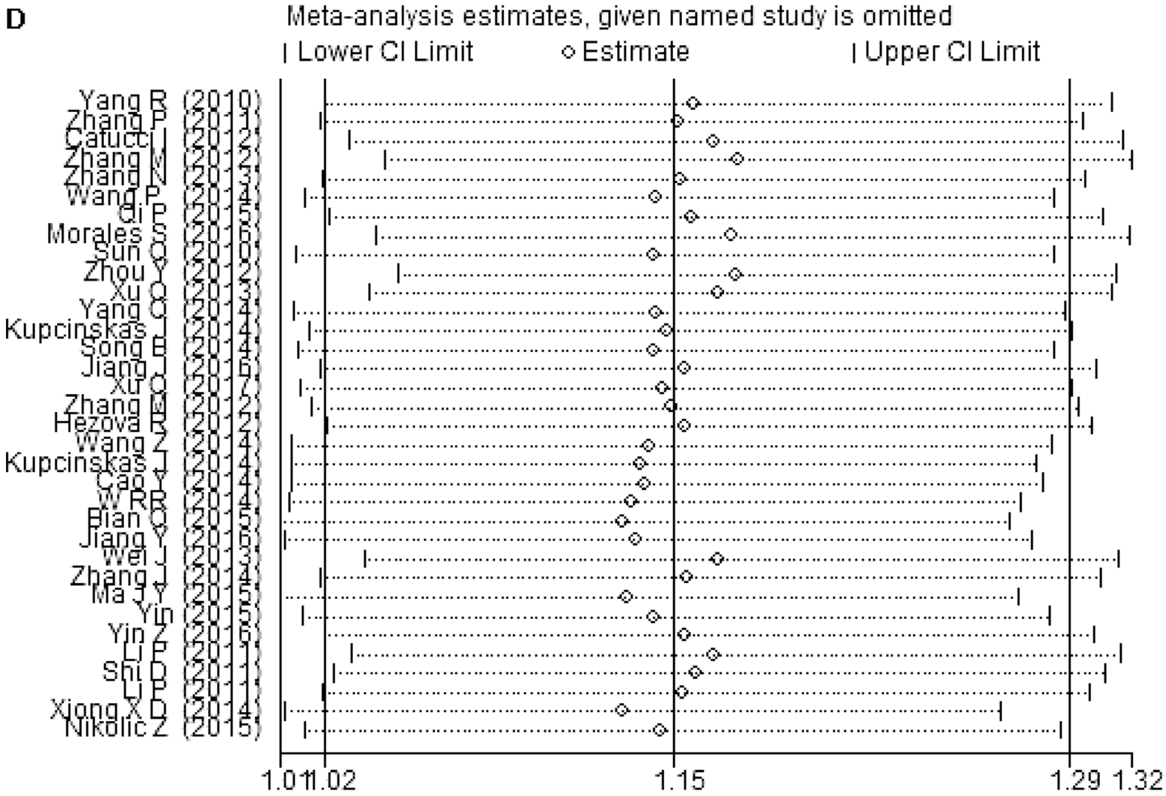

E

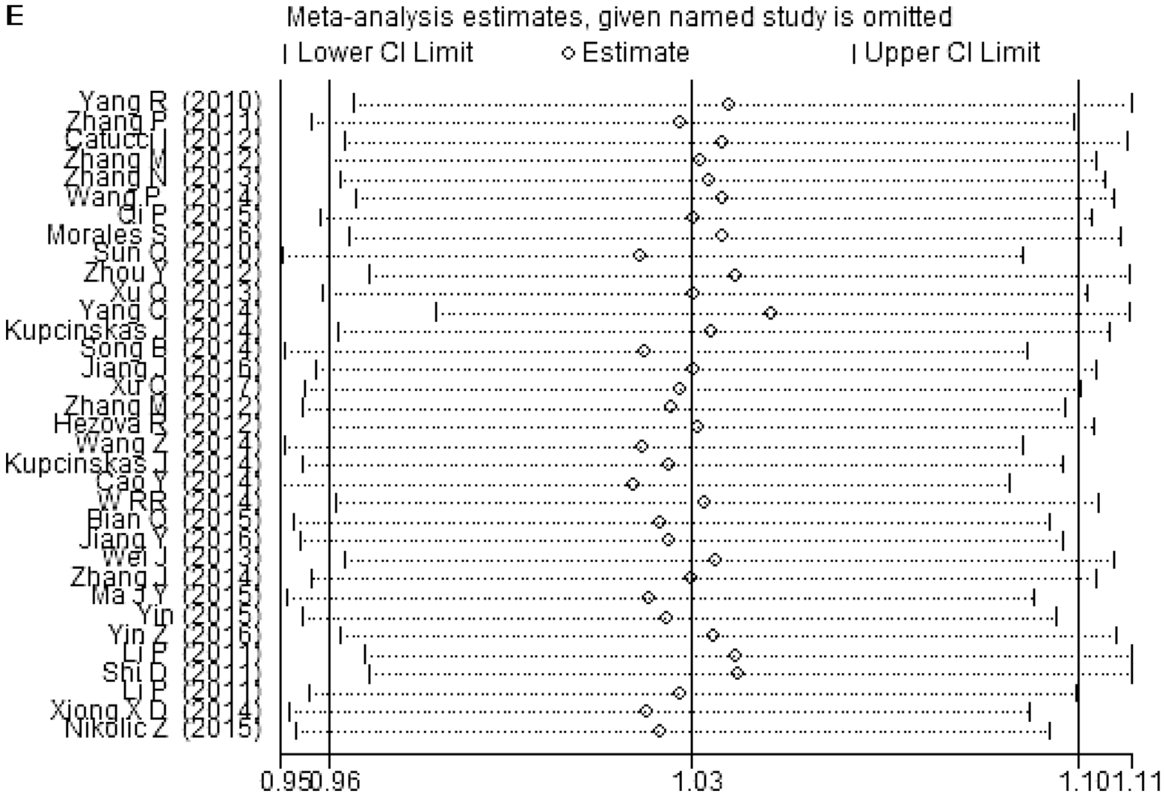

Supplementary Figure 1: Sensitivity analysis of the summary OR on the association between rs895819 and cancer risk. The association of rs895819 with cancer risk was computed by omitting each study in turn in heterozygous (**A**), homozygous (**B**), dominant (**C**), recessive (**D**) and additive model (**E**). The two ends of the dotted lines represent the 95% CI of the OR.

Supplementary Table 1: Stratified analysis of the association between miR-27a polymorphisms and cancer risk

Groups	*n*[a]	Heterogenous		Homogenous		Dominant		Recessive		Additive	
		OR (95% CI)[b]	*P*[c]	OR (95% CI)[b]	*P*[c]	OR (95% CI)[b]	*P*[c]	OR (95% CI)[b]	*P*[c]	OR (95% CI)[b]	*P*[c]
Breast cancer	8	0.93 (0.77–1.11)	0.002	0.88 (0.76–1.02)	0.834	0.91 (0.80–1.05)	0.052	0.90 (0.77–1.05)	0.351	**0.91 (0.86–0.97)**	0.682
Ethnic											
Asian	5	0.96 (0.66–1.40)	0.002	1.02 (0.78–1.33)	0.933	0.98 (0.75–1.28)	0.040	0.94 (0.70–1.26)	0.255	0.98 (0.86–1.10)	0.545
Caucasian	3	**0.86 (0.76–0.99)**	0.221	**0.83 (0.70–0.99)**	0.563	**0.85 (0.77–0.94)**	0.485	0.89 (0.73–1.08)	0.294	**0.89 (0.82–0.96)**	0.926
Digestive system	19	0.96 (0.81–1.15)	< 0.001	**1.20 (1.01–1.43)**	< 0.001	1.03 (0.89–1.19)	< 0.001	**1.23 (1.06–1.43)**	0.004	1.07 (0.97–1.17)	< 0.001
Ethnic											
Asian	16	0.98 (0.80–1.19)	< 0.001	1.19 (0.98–1.45)	< 0.001	1.04 (0.88–1.23)	< 0.001	**1.21 (1.02–1.43)**	0.002	1.07 (0.97–1.19)	< 0.001
Caucasian	3	0.87 (0.71–1.07)	0.511	1.23 (0.88–1.71)	0.365	0.93 (0.76–1.13)	0.443	1.31 (0.95–1.79)	0.381	1.01 (0.88–1.17)	0.372
Digestive tracts	18	0.95 (0.79–1.15)	< 0.001	**1.20 (1.00–1.44)**	< 0.001	1.02 (0.88–1.19)	< 0.001	**1.24 (1.06–1.45)**	0.003	1.06 (0.97–1.17)	< 0.001
Ethnic											
Asian	15	0.97 (0.78–1.19)	< 0.001	1.19 (0.97–1.47)	< 0.001	1.04 (0.87–1.24)	< 0.001	**1.22 (1.02–1.46)**	0.001	1.07 (0.96–1.20)	< 0.001
Caucasian	3	0.87 (0.71–1.07)	0.511	1.23 (0.88–1.71)	0.365	0.93 (0.76–1.13)	0.443	1.31 (0.95–1.79)	0.381	1.01 (0.88–1.17)	0.372
UADT cancer	10	0.95 (0.73–1.25)	< 0.001	0.98 (0.76–1.27)	0.001	0.97 (0.77–1.22)	< 0.001	1.04 (0.83–1.30)	0.006	0.99 (0.87–1.13)	< 0.001
Ethnic											
Asian	9	0.98 (0.73–1.31)	< 0.001	0.97 (0.73–1.28)	0.001	0.99 (0.77–1.27)	< 0.001	1.01 (0.79–1.29)	0.004	1.00 (0.86–1.16)	< 0.001
Caucasian	1	0.76 (0.56–1.03)	N/A[d]	1.09 (0.65–1.84)	N/A[d]	0.81 (0.60–1.09)	N/A[d]	1.25 (0.75–2.06)	N/A[d]	0.92 (0.74–1.16)	N/A[d]
Gastric cancer	8	0.94 (0.66–1.34)	< 0.001	1.00 (0.74–1.37)	0.001	0.97 (0.72–1.31)	< 0.001	1.08 (0.83–1.40)	0.008	1.00 (0.84–1.19)	< 0.001
Ethnic											
Asian	7	0.97 (0.65–1.45)	< 0.001	0.98 (0.69–1.41)	0.001	1.00 (0.71–1.39)	< 0.001	1.04 (0.77–1.41)	0.004	1.01 (0.83–1.23)	< 0.001
Caucasian	1	0.76 (0.56–1.03)	N/A[d]	1.09 (0.65–1.84)	N/A[d]	0.81 (0.60–1.09)	N/A[d]	1.25 (0.75–2.06)	N/A[d]	0.92 (0.74–1.16)	N/A[d]
Colorectal cancer	8	0.97 (0.78–1.20)	0.005	**1.56 (1.31–1.85)**	0.758	1.10 (0.94–1.29)	0.067	**1.53 (1.30–1.79)**	0.582	**1.19 (1.09–1.30)**	0.351
Ethnic											
Asian	5	0.96 (0.72–1.28)	0.001	**1.61 (1.33–1.94)**	0.866	1.12 (0.92–1.38)	0.027	**1.56 (1.31–1.85)**	0.645	**1.21 (1.10–1.34)**	0.316
Caucasian	3	0.97 (0.74–1.27)	0.995	1.31 (0.75–2.29)	0.194	1.03 (0.80–1.33)	0.683	1.33 (0.76–2.33)	0.171	1.08 (0.89–1.32)	0.357

[a]Number of comparisons.
[b]The crude OR and 95% CI were calculated based on the genotype frequencies.
[c]*P* value of *Q*-test for heterogeneity analysis.
[d]N/A: Not available.
